# A case report of malignant hypertension in a young woman

**DOI:** 10.1186/s12882-016-0291-x

**Published:** 2016-07-07

**Authors:** Andrea Michelli, Stella Bernardi, Andrea Grillo, Emiliano Panizon, Matteo Rovina, Moreno Bardelli, Renzo Carretta, Bruno Fabris

**Affiliations:** Department of Medical, Surgical and Health Sciences, University of Trieste, Cattinara Teaching Hospital, Strada di Fiume 447, Trieste, Italy

**Keywords:** Malignant hypertension, Acute kidney injury, Renin-angiotensin-aldosterone system, Young woman

## Abstract

**Background:**

Malignant hypertension is a condition characterized by severe hypertension and multi-organ ischemic complications. Albeit mortality and renal survival have improved with antihypertensive therapy, progression to end-stage renal disease remains a significant cause of morbidity and mortality. The underlying cause of malignant hypertension, which can be primary or secondary hypertension, is often difficult to identify and this can substantially affect the treatment outcomes, as we report here.

**Case presentation:**

A 33-year-old woman presented with severe hypertension and acute renal failure. Initial evaluation demonstrated hyperreninemia with hyperaldosteronism and a possible renal artery stenosis at the contrast-enhanced CT scan. Although this data suggested the presence of a secondary form of hypertension, further exams excluded our first diagnosis of renal artery stenosis. Consequently, the patient did not undergo renal angiography (and the contrast media infusion associated with it), but she continued to be medically treated to achieve a tight blood pressure control. Our conservative approach was successful to induce renal function recovery over 2 years of follow-up.

**Conclusion:**

This case highlights the difficulty in differentiating between primary and secondary forms of malignant hypertension, particularly when the patient presents with acute renal failure. Clinicians should consider renal artery ultrasound as a first level diagnostic technique, given that the presentation of primary malignant hypertension can often mimic a renal artery stenosis. Secondly, adequate control of blood pressure is essential for kidney function recovery, although this may require a long time.

## Background

Malignant hypertension is a condition characterized by severe hypertension and multi-organ ischemic complications [1]. Incidence of malignant hypertension has remained stable over the years, although mortality and renal survival have improved with the introduction of antihypertensive therapy. However, progression to end-stage renal disease remains a significant cause of morbidity and mortality [2]. The underlying cause of malignant hypertension can be primary or secondary hypertension, and identification of the latter is mandatory for choosing the correct treatment in order to control blood pressure and improve end-organ damage. However, correct diagnosis can be challenging [3]. This case highlights the difficulty in differentiating between primary and secondary hypertension, particularly when the patient presents with acute renal failure.

## Case presentation

A 33-year-old woman was referred to our Internal Medicine Department by her GP after the recent diagnosis of severe hypertension. While the diagnosis of hypertension dated back to the day before, its onset was actually unknown, as the patient had no memory of having ever measured her blood pressure before, consistent with the low awareness that young adults have of their hypertension [4]. During the visit she complained of fatigue. Otherwise, her medical and family histories were unremarkable. She used to smoke no more than 5 cigarettes per day, did not take any prescription or over-the-counter medications and denied the use of recreational drugs, as confirmed by the negativity of the urine drug screening. On admission, her blood pressure was 240/140 mmHg, but her other vital signs were normal and physical examination was unremarkable. Initial laboratory studies identified the presence of renal failure (creatinine 2.11 mg/dL), hypokalemia (potassium 2.45 mEq/L), and anemia with thrombocytopenia (hemoglobin 10.6 g/dL, platelets 113.000/microm^3^), which were likely to be hemolytic as LDH was elevated (572 U/L). There was also an elevated CRP level of 170 mg/L. As for end-organ damage, renal failure was associated with a proteinuria of 1.9 g over 24 h, while ultrasound revealed 2 normal-sized kidneys with echogenic parenchyma. The ECG showed signs of left ventricular hypertrophy, which was confirmed by echocardiography, as the interventricular septum thickness measured 19 mm and LV mass/BSA was 232 g/m^2^. The left ventricular ejection fraction was 50 %, and there was no aortic coarctation. Retinal examination revealed grade III hypertensive retinopathy, showing the presence of malignant hypertension, and antihypertensive drugs were promptly administered.

Given that the clinical characteristics suggestive of secondary causes of hypertension include early (i.e. < 30 years) and sudden onset of hypertension in patients without other risk factors, blood pressure levels higher than 180/110 mmHg, and presence of target end-organ damage [5], our next exams were aimed at excluding secondary causes of hypertension. These analyses showed that our patient had a hyperreninemia with a secondary hyperaldosteronism (renin 266.4 microUI/mL, aldosterone 38.1 ng/dL), which could be due to the presence of a renovascular disease, a renin-secreting tumor, or a scleroderma renal crisis [6]. This last hypothesis was however excluded by the absence of circulating autoantibodies, as well as the absence of other clinical and/or laboratory features suggestive of immunological disorders. Moreover, a week after the start of the antihypertensive therapy, not only CRP, but also hemoglobin, platelets, and LDH normalized, so that we ruled out also other conditions causing renal failure with thrombotic microangiopathy and secondary hypertension, such as the hemolytic uremic syndrome (HUS) and the thrombotic thrombocytopenic purpura (PTT) [7].

**Fig. 1:**
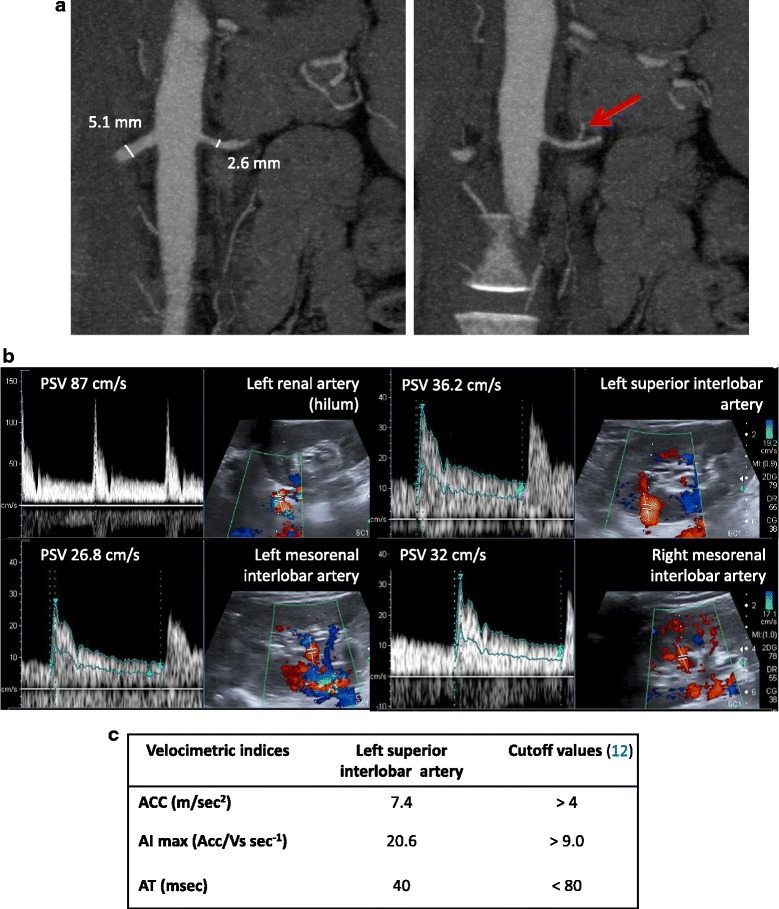
**a** Abdomen CT scan image. The red arrow indicates the suspected left renal artery stenosis. **b** Renal echocolordoppler images and velocimetric indices. PSV, peak systolic velocity; ACC, maximal systolic acceleration, AI_max_, maximal acceleration index; AT, acceleration time

Given the severity of the case, the diagnosis of any underlying curable cause of the patient hypertension could not be overlooked. At that stage, taking into account the laboratory exams, we needed to rule out several possible causes of malignant hypertension, including renal artery stenosis, and other insidious diseases such as phaeocromocytomas [8], lymphomas, and other renin-secreting masses [9]. For this reason, according to current guidelines [10, 11], the patient underwent a contrast enhanced computed tomography (CT) of the abdomen. This exam did not show any suspicious masses. Nevertheless, it visualized a stenotic left renal artery (Fig. 1a), suggesting that our patient could have a fibromuscular dysplasia causing renal artery stenosis. Despite most of our results were already strongly suggestive of renal artery stenosis, before prescribing any angiography with angioplasty, we requested a renal duplex ultrasound exam. The analysis of blood flow velocity, which was performed at the renal hilum as well as the intraparenchymal arteries, showed a normal hemodynamic pattern (Fig. 1b). Moreover, both proximal and distal velocimetric indices were normal (Fig. 1c). In particular, the maximal acceleration index, whose sensitivity is 93 % and specificity is 84 % [12], was greater than 9 s^-1^, at all the sites. So, the renal duplex ultrasound did not confirm -to our surprise- the suspected renal artery stenosis.

**Fig. 2:**
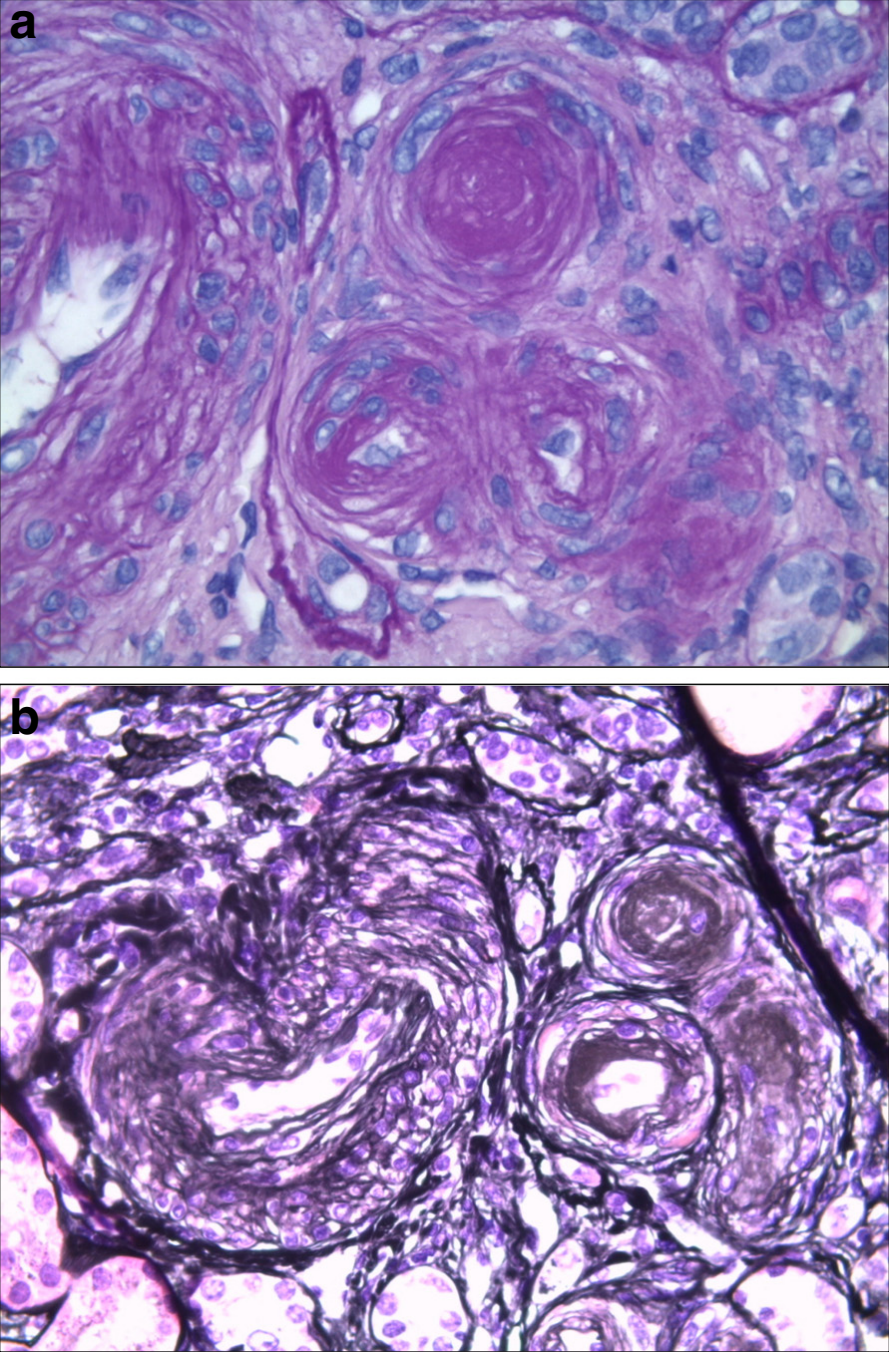
**a-b** Representative images of kidney biopsy pathology, where vessel wall thickening with aspects of onionskin hyperplasia, endothelial layer detachment, and intraluminal platelet thrombosis with partial or complete obstruction of the vessel lumina can be seen

Given this result, the renal angiography was put on hold and the patient was treated with medical therapy only, achieving blood pressure normalization over a few weeks. Nevertheless, the persistence of the renal failure, despite blood pressure normalization, led us to perform a kidney biopsy in order to exclude primary renal diseases. Kidney biopsy showed relative sparing of glomeruli with predominant vascular damage (Fig. 2). This finding led us to the final diagnosis of malignant hypertension complicating an underlying primary (essential) hypertension with thrombotic microangiopathy.

**Fig. 3:**
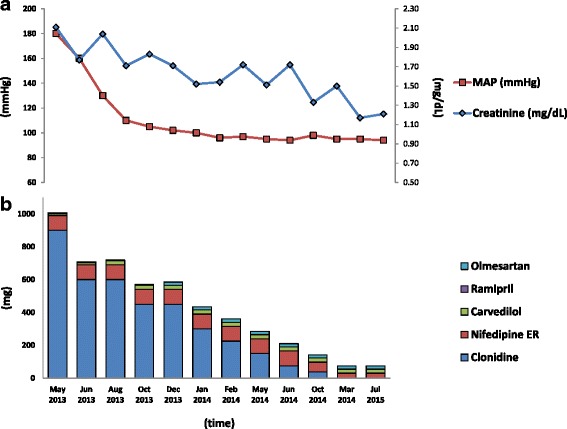
Mean arterial pressure and creatinine normalization. **a** antihypertensive therapy reduction. **b** over the 2-year follow-up

In this case antihypertensive therapy was able to successfully reduce blood pressure and induce end-organ damage recovery (Fig. 3). The echocardiography showed that after 1 year from the start of the therapy the interventricular septum thickness was of 11 mm, the LV mass/BSA was of 61 g/m^2^, and that the ejection fraction was of 74 %. The retinal examination did not show any cotton wool spots or flame hemorrhages. Proteinuria disappeared 4 months after hospitalization and renal function progressively ameliorated over the following 2 years (Fig. 3), reflecting a slow recovery process that is likely to include vascular remodeling [6].

## Conclusions

This young woman’s presentation, marked by malignant hypertension with renal failure, was a diagnostic challenge. On one hand, the clinical presentation, the hyperreninemia with a secondary hyperaldosteronism were suggestive of a secondary form of hypertension, which at the contrast-enhanced CT scan seemed to be that of a renal artery stenosis. On the other hand, the renal duplex ultrasound exam was normal. Had there been a renal artery stenosis, the angiography (with angioplasty) might have been essential to successfully treat both hypertension and renal failure. On the contrary, if this had not been the case, unnecessary contrast media administration could have prevented renal recovery. In the end, we relied on the sensitivity [12] of the renal duplex ultrasound exam and decided to avoid the angiography.

This case highlights the difficulty in differentiating between primary and secondary hypertension in cases of malignant hypertension. More than half of the cases of malignant hypertension are in fact due to essential hypertension [13]. Moreover, be it essential or secondary, the clinical presentation of malignant hypertension can be the same. In addition, if the clinical presentation does not help discriminate, laboratory might not help either, in particular when it comes to the measurement of renin and aldosterone. Malignant hypertension is in fact typically associated with an activation of the renin-angiotensin-aldosterone system (RAAS) [14]. Although the exact mechanism of malignant hypertension is unknown, several studies have implicated the RAAS as a key factor to its pathogenesis. The severe elevation of blood pressure would in fact lead to RAAS activation through microvascular damage and renovascular ischemia, and the increased production of Angiotensin II would in turn further increase blood pressure, leading to malignant hypertension [14]. In this setting, it is not unusual to find also other changes due to endothelial dysfunction [15], which might explain the transient microangiopathic hemolysis and increased CRP of our case.

Secondly, this case underlines the importance of performing the renal artery duplex ultrasound as the first-line (screening) exam when a renal artery stenosis is suspected, before considering the renal angiography with angioplasty, which is the reference standard for the anatomic diagnosis and treatment of renal artery stenosis [10]. Given that angiography is an invasive procedure that carries a risk for serious complications, less invasive techniques are advocated for the initial work-up of patients with suspected renal artery stenosis [12]. For this reason, the European consensus on the diagnosis and management of fibromuscular dysplasia suggests starting the patient evaluation with renal duplex ultrasound and then confirming the diagnosis with a CT-angiography prior to angioplasty. As compared to computed tomography, whose sensitivity has not always been found sufficient to rule out a renovascular disease [16], the renal duplex ultrasound exam, by the assessment of intrarenal velocimetric indices, has a high sensitivity and a high negative predictive value [12]. On the other hand, the results of the renal duplex ultrasound exam can be suboptimal if it is performed on obese patients, when apnea is difficult or impossible, and where local expertise is poor [10]. Nevertheless, in our case, given the patient’s slender figure and compliance, as well as local expertise, we took a step backward and decided to schedule a renal duplex ultrasound before the angiography. Contrary to the CT scan, the renal duplex ultrasound turned out to be negative. Therefore, given that there had not been technical biases hindering the diagnostic accuracy of the exam, we based our next decision on the high sensitivity and negative predictive value of the duplex ultrasound. This helped avoid the angiography as well as the additional contrast media administration that could have affected negatively the renal recovery [17].

Despite the fact that over the last 40 years the incidence of malignant hypertension has not changed and remains 2-3/100,000/year, its prognosis has improved significantly [13], which can be ascribed to the introduction of modern antihypertensive drugs and a better blood pressure control. Likewise, also renal prognosis has improved, and the probability of renal survival is 84 % and 72 % after 5 and 10 years of follow-up, respectively [18]. In the end, our case confirms that a tight control of blood pressure during follow-up is one of the main predictors of renal outcome in patients with malignant hypertension [18].
